# An improved U-net and attention mechanism-based model for sugar beet and weed segmentation

**DOI:** 10.3389/fpls.2024.1449514

**Published:** 2025-01-13

**Authors:** Yadong Li, Ruinan Guo, Rujia Li, Rongbiao Ji, Mengyao Wu, Dinghao Chen, Cong Han, Ruilin Han, Yongxiu Liu, Yuwen Ruan, Jianping Yang

**Affiliations:** ^1^ College of Big Data, Yunnan Agricultural University, Kunming, China; ^2^ Modern Educational Technology Center, Yunnan Agricultural University, Kunming, China; ^3^ School of Information Technology and Artificial Intelligence, Zhejiang University of Finance and Economics, Hangzhou, China; ^4^ College of Big Data, Baoshan University, Baoshan, China; ^5^ College of Economics and Management, Yunnan Agricultural University, Kunming, China; ^6^ College of plant protection, Yunnan Agricultural University, Kunming, China; ^7^ Faculty of Sciences, University of Copenhagen, Copenhagen, Denmark; ^8^ The Key Laboratory for Crop Production and Smart Agriculture of Yunnan Province, Yunnan Agricultural University, Kunming, China

**Keywords:** semantic segmentation, UNET, deep learning, MaxViT, CBAM, attention mechanism, image processing, multi-scale features

## Abstract

**Introduction:**

Weeds are a major factor affecting crop yield and quality. Accurate identification and localization of crops and weeds are essential for achieving automated weed management in precision agriculture, especially given the challenges in recognition accuracy and real-time processing in complex field environments. To address this issue, this paper proposes an efficient crop-weed segmentation model based on an improved UNet architecture and attention mechanisms to enhance both recognition accuracy and processing speed.

**Methods:**

The model adopts the encoder-decoder structure of UNet, utilizing MaxViT (Multi-Axis Vision Transformer) as the encoder to capture both global and local features within images. Additionally, CBAM (Convolutional Block Attention Module) is incorporated into the decoder as a multi-scale feature fusion module, adaptively adjusting feature map weights to enable the model to focus more accurately on the edges and textures of crops and weeds.

**Results and discussion:**

Experimental results show that the proposed model achieved 84.28% mIoU and 88.59% mPA on the sugar beet dataset, representing improvements of 3.08% and 3.15% over the baseline UNet model, respectively, and outperforming mainstream models such as FCN, PSPNet, SegFormer, DeepLabv3+, and HRNet. Moreover, the model’s inference time is only 0.0559 seconds, reducing computational overhead while maintaining high accuracy. Its performance on a sunflower dataset further verifies the model’s generalizability and robustness. This study, therefore, provides an efficient and accurate solution for crop-weed segmentation, laying a foundation for future research on automated crop and weed identification.

## 1 Introduction

With the continuous growth of the global population and increasing demand for food, enhancing agricultural productivity has become a core objective in modern agricultural development. Precision agriculture, as a novel management approach, aims to achieve precise crop management through technological methods, maximizing yield and resource use efficiency (Li et al., 2024; Chen et al., 2024). Among the critical factors affecting crop yield and quality, weed management is particularly impactful. Traditional manual weeding is not only time-consuming and labor-intensive but also poses a risk of damaging crops. Consequently, advancing intelligent agricultural technology, particularly in automated weed detection and segmentation, is urgently needed. The use of agricultural chemicals, such as herbicides and pesticides, is a common approach to managing weed invasions. Currently, weed control primarily relies on uniform pesticide spraying, where herbicides are applied at the same dosage to weeds indiscriminately. This method fails to distinguish between crops and weeds, resulting in significant pesticide wastage, soil and water pollution, and negative impacts on farmland productivity and crop growth. To reduce pesticide waste and improve pesticide utilization, research on precision variable-rate pesticide spraying based on weed detection is essential (Jin et al., 2022; Kamath et al., 2022; Simonyan and Zisserman, 2014).

In recent years, deep learning-based image segmentation techniques have been widely applied to crop and weed segmentation tasks in precision agriculture, emerging as a key technology for intelligent agriculture. For example, the introduction of Fully Convolutional Networks (FCNs) in 2015 (Long et al., 2015) marked a new era for semantic segmentation. This network structure achieved a transition from image-level to pixel-level classification, laying the foundation for subsequent semantic segmentation models. Later, Google’s DeepLab series (Chen L. et al., 2017; Chen L. C. et al., 2017) improved segmentation accuracy and model efficiency by enlarging the receptive field and introducing an encoder-decoder structure. PSPNet (Zhao et al., 2017) proposed a pyramid pooling module that, built upon the FCN network, integrates contextual features from various scales, facilitating global context aggregation. In 2019, Google introduced the DeepLabV3+ model (Chen et al., 2018), which incorporated an encoder-decoder structure into V3 and used depthwise separable convolutions to reduce model parameters and enhance accuracy. However, these conventional semantic segmentation models exhibit limitations when processing agricultural images, particularly in dealing with complex backgrounds and multi-scale targets. These models especially struggle with accurately segmenting weeds at the boundaries when crops and weeds overlap, leading to suboptimal control outcomes.

A hybrid research field combining metaheuristic algorithms with machine learning methods has emerged recently. This novel direction leverages the advantages of machine learning and swarm intelligence, showing exceptional performance across various applications (Bacanin et al., 2021; Malakar et al., 2020; Zivkovic et al., 2022; Dobrojevic et al., 2023; Jovanovic et al., 2023; Derrac et al., 2011) and promising breakthroughs in tasks such as pattern recognition and image segmentation. Additionally, self-attention-based Transformer structures (Lyu et al., 2019), originally successful in natural language processing, have demonstrated superior performance when extended to computer vision (Han et al., 2022; Bai et al., 2022; Deng et al., 2022), opening up new research avenues in this field. For instance, the SegFormer model (Xie et al., 2021) combines Transformers with a lightweight multilayer perceptron decoder, providing stronger long-range feature dependencies and spatial transformations compared to CNNs. The global context modeling achieved through self-attention enables the extraction of more comprehensive image feature vectors, offering a direct and efficient solution.

UNet (Ronneberger et al., 2015), a classical model in semantic segmentation, introduced the encoder-decoder structure and employed skip connections to preserve detail features, showing remarkable performance in medical image segmentation. Its capability to capture boundary information and details has attracted significant attention. However, directly applying UNet to agricultural image segmentation often yields limited performance. Recently, improved UNet architectures have been widely adopted for crop-weed segmentation research. For example, Ma et al. (2019) proposed a SegNet-based method for rice and broadleaf weed segmentation, and Brilhador et al. (2019) applied an enhanced UNet to segment crops and weeds at the pixel level. While these improvements have enhanced segmentation accuracy to some extent, there remains significant room for improvement in boundary information extraction and handling complex backgrounds. To address these issues, this study proposes an efficient crop-weed segmentation model based on an improved UNet with attention mechanisms. Our main innovation lies in incorporating the MaxViT (Multi-Axis Vision Transformer) (Tu et al., 2022) and CBAM (Convolutional Block Attention Module) modules (Woo et al., 2018), enhancing UNet’s feature extraction capability and segmentation accuracy by integrating multi-scale features and boundary information. Compared with existing UNet improvements and mainstream Transformer segmentation models, our proposed model achieves a better balance between computational efficiency and segmentation accuracy. The primary contributions of this paper include:

1. Proposing an improved UNet architecture combined with MaxViT and CBAM modules, enhancing segmentation accuracy and computational efficiency.
2. Conducting extensive experiments to validate the superior performance of the improved model in crop-weed segmentation tasks.
3. Testing the model across various datasets to demonstrate its generalization and robustness.
4. Providing new insights and technical support for weed management in precision agriculture.

Based on the above research background and innovations, the structure of this paper is organized as follows: Section 2 details the design and implementation of the crop-weed segmentation model combining improved UNet and attention mechanisms; Section 3 describes the experimental setup and analyzes the results, including comparison, ablation, and generalization experiments; Section 4 discusses the advantages, limitations, and future directions of this research.

## 2 Materials and methods

### 2.1 Overall architecture

This study introduces an innovative model that combines a vision transformer with the traditional UNet network to improve the precision and robustness of crop-weed segmentation. The UNet architecture employs a U-shaped encoder-decoder structure, where the encoder extracts features through successive convolution and pooling layers, and the decoder reconstructs these features to match the input image resolution. While UNet’s convolution-based encoder is highly effective in capturing local features, it falls short in capturing global context, particularly in complex backgrounds and texture-rich scenes, which limits its segmentation accuracy. To address these limitations, we utilize MaxViT as the encoder for UNet. MaxViT is a high-efficiency, multi-axis vision transformer that combines both local and global attention mechanisms, enabling it to capture interactions across local and global features at each stage. Unlike traditional UNet encoders, MaxViT is more adept at capturing long-range dependencies and global context, allowing the model to better focus on subtle crop-weed distinctions and enhancing its adaptability to varying crop types and complex environments.

**Figure 1:**
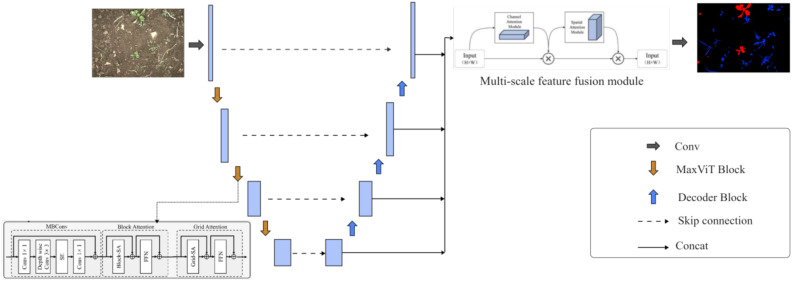
Overall structure of the improved model.

As illustrated in **Figure 1**, the modified model retains the UNet encoder-decoder framework, utilizing MaxViT to extract deep feature representations that are transferred to the decoder through skip connections, ensuring the retention of fine details and preserving feature resolution. The decoder comprises four stages, primarily tasked with restoring the image’s resolution. In the final stage, a CBAM module is applied as a multi-scale feature fusion mechanism, enhancing the model’s ability to represent small-scale crop and weed features and focus more precisely on these regions rather than background areas. This refinement improves the segmentation of crop and weed boundaries significantly. The uniqueness of our model lies in combining the encoder-decoder architecture with MaxViT and CBAM attention mechanisms. This integration not only boosts segmentation performance but also offers enhanced stability and generalization across diverse crop types and environmental conditions, demonstrating the model’s robustness and applicability in agricultural settings.

#### 2.1.1 MaxViT encoder

**Figure 2:**
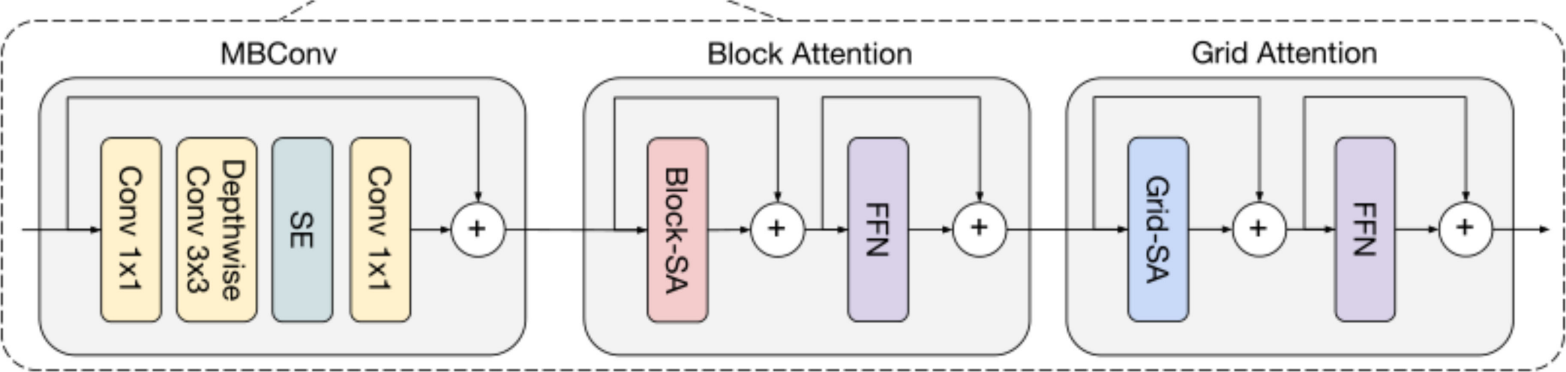
Working mechanism of MaxViT module.

The model employs a four-stage encoder to extract multi-stage features from sugar beet dataset images. Each stage’s resolution is half that of the previous feature map, with the number of channels doubled. These feature maps are connected to the decoder via skip connections. As shown in **Figure 2**, each stage consists of MaxViT modules, each containing MBConv, block attention mechanism, and grid attention mechanism. As shown in **Figure 1**, the feature maps generated by MBConv are input to the block attention module and the grid attention module. In crop and weed segmentation, MBConv differentiates between crops and weeds by precisely capturing local features. This structure effectively improves parameter efficiency and computational speed, making it suitable for real-time image processing on mobile or edge devices. The block attention mechanism focuses on feature aggregation within specific areas, enhancing the expressiveness of local regions by concentrating on small blocks, which helps the model to focus on details in small regions of crops and weeds, thereby distinguishing adjacent but different categories of objects. The grid attention mechanism operates at a global level, adjusting and enhancing feature expressions across the entire image, integrating information from the entire image, optimizing segmentation boundaries, and improving segmentation accuracy and consistency.

MaxViT uses the MBConv block as the primary convolutional operator. The MBConv block is formulated as:


(1) $$X_{MBCov}=X+\text{proj}\left(SE\left(DWConv_{3\times 3}\left({\text{Conv}}_{1\times 1}\left(\text{Norm}\left(X\right)\right)\right)\right)\right)$$

where *X* and $X_{MBConv}$ are the input and output feature maps, respectively. Norm refers to the batch norm and ${\text{Conv}}_{1\times 1}$ is the convolution operation with a 1×1 kernel size. $DWConv_{3\times 3}$ denotes the depthwise convolution with a 3×3 kernel size, *SE* is the squeeze-excitation (SE) layer and proj is a convolution operation with a 1×1 kernel size to reduce the number of channels.

In MaxViT blocks, all the attention operators used relative attention defined in Equation 2: RelAttention:


(2) $$RelAtention\left(Q,K,V\right)=\text{softmax}\left(\frac{QK^{T}}{\sqrt{d}}+B\right)V$$

where $Q,K,V\in R^{\left(H\times W\right)\times C}$ are the query, key, and value matrices, (H×W)×C denotes the dimensions of these matrices, where *H* and *W* are the height and width of the feature map,respectively,and *C* is the number of channels. *d* is the hidden dimension and *B* refers to a learned static location-aware matrix. In the block attention module, the hyperparameter *P* was defined initially to divide the input feature map $X\in R^{H\times W\times C}$ into $\frac{H}{P}$ nonover - lapping blocks of size P×P . The shape of the feature map is described in Equation 3 below:


(3) $$\left(H,W,C\right)\to \left(\frac{H}{P}\times P,\frac{W}{P}\times P,C\right)\to \left(\frac{HW}{P^{2}}\times P,P^{2},C\right)$$

Then, the relative attention on the second dimension was performed, meaning that the local characteristic of the soybean field within the block was obtained. The forward of the grid attention is described in Equation 4 below:


(4) $$\begin{array}{c} X_{Block}=X_{MBConv}\\ +UnBlock\left(RelAtention\left(Block\left(LN\left(X_{MBCon}\right)\right)\right)\right) \end{array}$$

where *LN* denotes the layer normalization and Block(·) and UnBlock() are the block partition and reverse block partition, respectively. Similarly, the feature map was divided into G lattices of size $\frac{H}{G}\times \frac{W}{G}$ by the hyperparameter *G* and the shape of the feature map is described in Equation 5 below:


(5) $$\left(H,W,C\right)\to \left(G\times \frac{H}{G},G\times \frac{W}{G},C\right)\to \left(G^{2},\frac{HW}{G^{2}},C\right)$$

The grid attention module was globally concerned with soybean canopy pixels and context information in sparse, uniform lattices covering the entire 2D space. The calculation process of the grid attention module was formulated as follows:


(6) $$X_{Grid}=X_{\text{Block}}+UnGrid\left(\text{RelAttention}\left(\text{Grid}\left(LN\left(X_{\text{B l o c k}}\right)\right)\right)\right)$$

Where Grid(.) and UnGrid(.) denote the grid partition and reverse grid partition, respectively.

#### 2.1.2 Multi-scale feature fusion module

Crops and weeds typically exhibit complex edge and texture features, requiring high-resolution detail information for accurate segmentation. As the soil serves as the background and contains various textures and color variations, the model must have strong background suppression capabilities. The traditional U-Net decoder leverages skip connections to obtain features from the encoder. However, due to insufficient fusion of features across layers, the decoding process is often limited to shallow, localized features, making it challenging to comprehensively incorporate multi-scale information. This limitation hinders segmentation performance in complex scenes. To enhance the model’s expressive capability, the proposed model’s multi-scale feature fusion module is designed to integrate both high-level and low-level features from all decoding stages. Unlike the traditional U-Net decoder, which simply up-samples layer by layer, the multi-scale feature fusion module processes features at different scales, improving the model’s ability to recognize objects of varying sizes and shapes. As shown in **Figure 1**, in the multi-scale feature fusion module, the output feature maps from the four decoder stages are first concatenated. The importance of each feature for crop and weed segmentation is not always the same. The CBAM (Convolutional Block Attention Module) introduces an adaptive feature weighting and selection mechanism into the deep learning model. It enhances important features and suppresses less important ones through channel attention and spatial attention modules. This enables the model to better focus on critical detail features, enhancing the edge information of crops and weeds while suppressing the interference from background soil.

**Figure 3:**
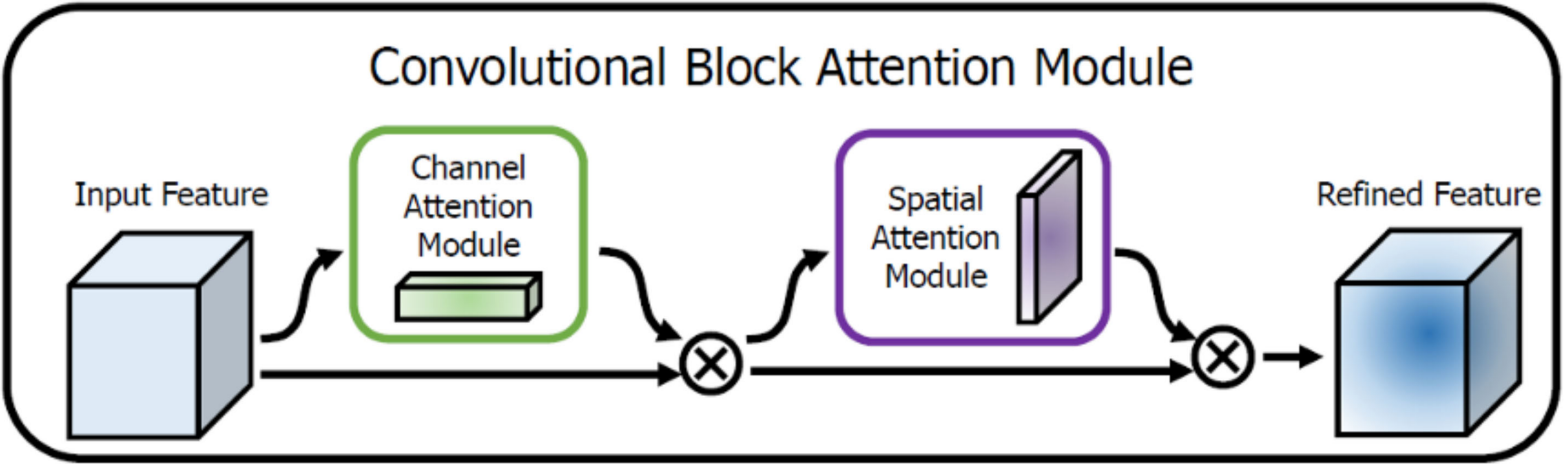
Working mechanism of CBAM module.

The CBAM module consists of two sequential sub-modules: a channel attention module and a spatial attention module. The channel attention module adjusts the weights of feature map channels to better utilize global and local contextual information. Simultaneously, the spatial attention module enhances the feature representation at key locations in the image, especially for small weeds, further improving feature recognition capability. Additionally, due to its structural design, CBAM can be easily embedded into various deep learning models, further enhancing their performance and segmentation result (Zhan et al., 2022). **Figure 3** illustrates the specific working mechanism of CBAM, demonstrating its flexibility and accuracy in feature processing and segmentation tasks.

Channel Attention Module: This module utilizes global average pooling $P_{\text{avg}}\left(F\right)$ and global max pooling $P_{\text{max}}\left(F\right)$ to extract channel feature descriptors. It then employs a shared fully connected layer, denoted as $M_{\text{LP}}$ , t to learn inter-channel relationships. The results are normalized using a sigmoid function σ to generate feature weights for each channel. The expression for this module is:


(7) $$\begin{array}{c} M_{c}\left(F\right)=\sigma \left\{M_{\text{LP}}\left[P_{\text{avg}}\left(F\right)\right]+M_{\text{LP}}\left[P_{\text{max}}\left(F\right)\right]\right\}\\ =\sigma \left\{W_{1}\left[W_{0}\left(F_{\text{avg}}^{c}\right)\right]+W_{1}\left[W_{0}\left(F_{\text{max}}^{c}\right)\right]\right\} \end{array}$$

In this formula, $P_{\text{avg}}\;$ and $P_{\text{max}}\;$ represent the global average pooling and max pooling, respectively, over a small range, where $F_{\text{avg}}^{\text{c}}$ and $F_{\text{max}}^{c}$ are the average and maximum pooled channel features. Here, $M_{\text{LP}}$ is a simple feedforward neural network with a hidden layer size of *C*, and σ denotes the sigmoid function. Additionally, $W_{0}$ and $W_{1}$ are learnable weight matrices within $M_{\text{LP}};W_{0}$ reduces the channel dimension to capture essential features, and $W_{1}$ restores the original channel dimension to produce the attention weights for each channel (Song et al., 2023).

Spatial Attention Module: This module employs global average pooling and global max pooling to obtain spatial feature descriptors. It then learns the relationships between spatial locations using a 1 × 1 convolution. The results are normalized using the Sigmoid function to obtain weights for each spatial location, thereby weighting the spatial features (Pu et al., 2022). Its expression is:


(8) $$\begin{array}{c} M_{\text{S}}\left(F\right)=\sigma \left\{f^{7\times 7}\left[P_{\text{avg}}\left(F\right);P_{\text{max}}\left(F\right)\right]\right\}\\ =\sigma \left\{f^{7\times 7}\left[\left(F_{\text{avg}}^{\text{s}};F_{\text{max}}^{\text{s}}\right)\right]\right\} \end{array}$$

In this formula: $P_{\text{avg}}$ and $P_{\text{max}}$ denote global average pooling and max pooling, respectively, over spatial dimensions. Here, $F_{\text{avg}}^{\text{s}}$ and $F_{\text{max}}^{\text{s}}$ are the average and max pooled spatial features, while $f^{\left(7\times 7\right)}$ represents 7×7 aconvolution applied to capture broader spatial relationships.

#### 2.1.3 Loss function

For pixel-level image segmentation tasks, each pixel is treated as an independent classification problem. This study adopts the cross-entropy loss function, which is the most commonly used loss function in semantic segmentation. It compares the predicted probability distribution of each pixel with the true label distribution, measuring the accuracy of the prediction by calculating the difference between them. Specifically, for each pixel, the cross-entropy loss calculates the Kullback-Leibler divergence between the predicted probability distribution q and the true label distribution p. The formula is as follows:


(9) $$\text{CE}\left(\text{p},\text{q}\right)=-\sum_{\text{i}=1}^{\text{C}}{\text{p}}_{\text{i}}\text{log}\left({\text{q}}_{\text{i}}\right)$$

where C represents the number of classes, ${\text{p}}_{\text{i}}$ is the true label, and ${\text{q}}_{\text{i}}$ is the predicted probability.

### 2.2 Model evaluation metrics

The performance of the model is evaluated from three aspects: segmentation accuracy, parameter count, and efficiency. Segmentation accuracy is primarily assessed using pixel accuracy (PA), intersection over union (IoU), mean pixel accuracy (mPA), and mean intersection over union (mIoU). IoU measures the spatial overlap between the model’s predictions and the ground truth annotations, while PA represents the proportion of correctly predicted pixels to the total number of pixels. mIoU and mPA represent the average IoU and PA over the entire dataset, respectively, providing a comprehensive evaluation of the model’s performance. The complexity and efficiency of the model are also considered, measured by the number of parameters and inference time (the time it takes the model to make a prediction for a single sample). The optimal model is selected by balancing segmentation accuracy, parameter count, and efficiency. The calculation formulas for these evaluation metrics are as follows:


(10) $$PA=\frac{\sum_{i=0}^{K}{\text{p}}_{ii}}{\sum_{i=0}^{K}\sum_{j=0}^{K}{\text{p}}_{ij}}\times 100\%$$

(11) $$mPA=\frac{1}{K+1}\sum_{j=0}^{k}\frac{{\text{p}}_{ii}}{\sum_{j=0}^{k}{\text{p}}_{ij}}\times 100\%$$

(12) $$IoU=\sum_{i=0}^{K}\frac{{\text{p}}_{ii}}{\sum_{j=0}^{K}{\text{p}}_{ij}+\sum_{j=0}^{K}{\text{p}}_{ji}-{\text{p}}_{ii}}\times 100\%$$

(13) $$mIoU=\frac{1}{K+1}\sum_{i=0}^{K}\frac{{\text{p}}_{ii}}{\sum_{j=0}^{k}{\text{p}}_{ij}+\sum_{j=0}^{k}{\text{p}}_{ji}-{\text{p}}_{ii}}\times 100\%$$

where *K* indicates the number of different categories in the dataset, which was 3 in this study. ${\text{p}}_{ij}$ denotes the number of pixels for which target category *i* was predicted to be category *j. p_ji_* is the number of pixels for which target category *j* was predicted to be category *i*.

### 2.3 Dataset

**Figure 4:**
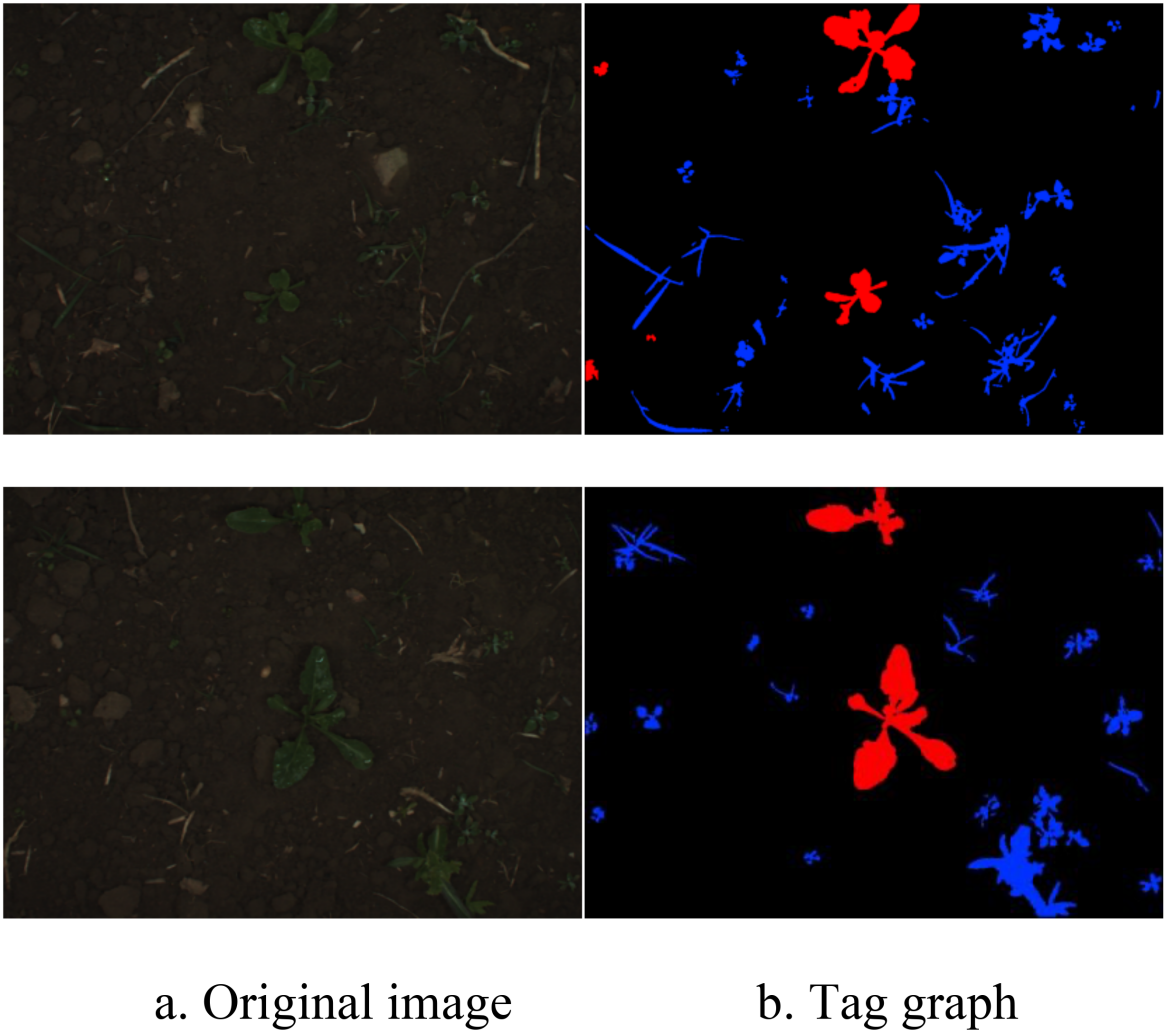
Examples of dataset. **(A)** Original image. **(B)** Tag graph.

The dataset used in this study consists of beet and weed images collected by the University of Bonn in Germany in 2016 (Chebrolu et al., 2017). The images were taken at a farm in Bonn, Germany, using a JAI AD-13 camera, covering different growth stages of beet. Due to the difficulty of pixel-level image annotation, the dataset contains a limited number of labeled images, with a total of 283 images. **Figure 4** shows some examples from the dataset. The images contain beets and various types of weeds, with red areas indicating beets, blue areas indicating weeds, and black areas indicating soil.

To effectively train and validate the model, the total samples were divided into a training dataset and a validation dataset at a ratio of 8:2. Given the small sample size of the original dataset, data augmentation methods such as flipping, rotation, scaling, cropping, and changing brightness and contrast were used to simulate variations in camera angles and lighting conditions during data collection, thereby enhancing the model’s generalization and robustness. These data augmentation techniques expanded the total sample from 283 images to 509 images, reducing the risk of overfitting (Lee et al., 2019).

## 3 Results

### 3.1 Experimental setup and network parameters

All models used in this study were run with GPU support, specifically utilizing an NVIDIA GeForce RTX 4090 graphics card. The models were implemented using the PyTorch deep learning framework. Considering hardware conditions and training effectiveness, the number of epochs was set to 200, with a batch size of 2 and a learning rate of 0.0001. The cross-entropy loss function was used. During the training process, to enhance the model’s initial performance and training efficiency, we utilized pre-trained weights based on transfer learning. The backbone networks of all models employed weights pre-trained on the PASCAL VOC2012 dataset. After loading the pre-trained weights, we fine-tuned the models to meet the specific requirements of our crop-weed task, thereby accelerating training speed and improving model performance.

**Figure 5:**
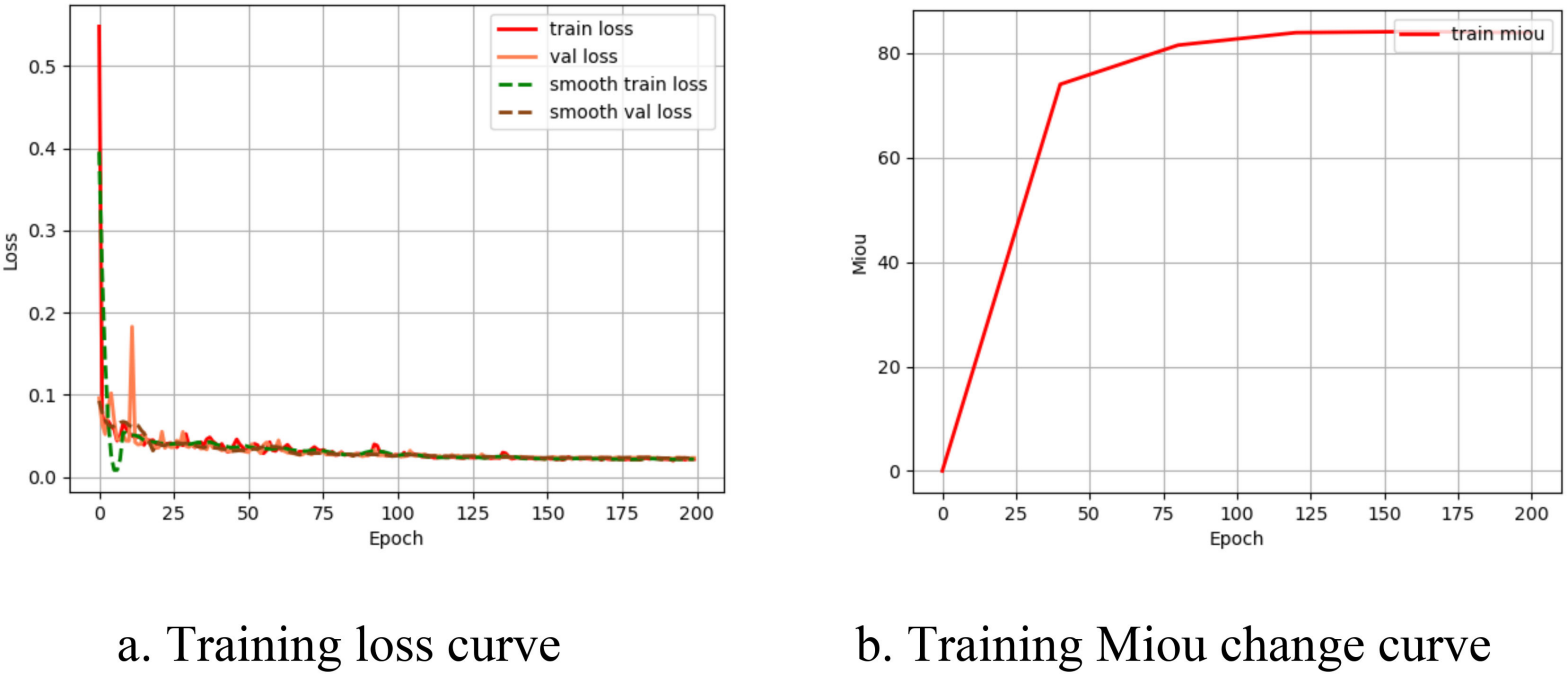
Model training process curve. **(A)** Training loss curve. **(B)** Training Miou change curve.

**Figure 5** shows the changes in loss values and mIoU over 200 epochs during the training process. Both the training loss and validation loss decreased rapidly at first, indicating fast learning. As training progressed, the rate of decrease slowed, indicating that the model began to stabilize and converge. The dashed lines represent smoothed trends, reducing noise and highlighting the overall downward trend, indicating good model performance. The close alignment of the training and validation losses suggests that the model generalizes well rather than overfitting. Temporary oscillations in the validation loss are normal and reflect the model’s response to different patterns in the validation set. By the time the curve reaches 200 epochs, it has reached a balanced state and completed convergence. The mIoU of the model increased with the number of iterations, reaching its maximum value around 200 epochs.

### 3.2 Comparative experiments

To verify the effectiveness of the proposed model, we conducted a comprehensive comparison with six other mainstream semantic segmentation models: FCN, PSPNet, SegFormer, DeepLabv3+, HRNet, and UNet. As shown in **Table 1**, our model demonstrates superior segmentation accuracy compared to other models, with improvements in mIoU of 19.6%, 28.33%, 5.05%, 5%, 8.21%, and 3.08%, respectively, and improvements in mPA of 17.1%, 28.16%, 4.34%, 5.27%, 6.77%, and 3.15%, respectively. Among them, FCN and PSPNet showed the poorest segmentation performance, indicating that simple convolutional layer stacking and direct multi-scale feature fusion are not effective for crop-weed segmentation. DeepLabv3+ struggled with weed feature extraction due to the lack of global feature extraction capability of the pure CNN encoder, leading to limited segmentation performance. HRNet’s segmentation performance was inferior to that of our proposed model, suggesting that its multi-resolution feature parallel processing is not effective enough for crop-weed segmentation. UNet achieved an mIoU of 81.20% and an mPA of 85.44%, outperforming SegFormer. This can be attributed to UNet’s skip connections, which continuously connect the multi-scale resolution feature maps of the encoder with the corresponding feature maps of the decoder, improving the segmentation accuracy of crops and weeds. Among all models, our proposed model achieved an mIoU of 84.28% and an mPA of 88.59%, due to the use of the MaxViT encoder based on Transformer and the integration of CBAM attention mechanism modules in the decoder stage to fuse multi-scale features, thereby enhancing the segmentation accuracy of crops and weeds. Compared to UNet, our model has a much smaller parameter size while maintaining similar segmentation accuracy. The PSPNet method has the smallest parameter count and the shortest inference time, but the lowest segmentation accuracy. Our model reduced inference time by 0.0252s, 0.0129s, 0.0496s, and 0.0276s compared to FCN, SegFormer, HRNet, and UNet, respectively. In summary, our model exhibits better segmentation performance, lower computational overhead, and achieves a balance between segmentation accuracy and inference speed.

**Table 1:** Overall comparison results of different methods.

Model	mIoU/%	mPA/%	Params/M	Inference time/s
FCN	64.68	71.49	21.835907	0.0811
PSPNet	55.95	60.43	2.375955	0.0343
SegFormer	79.23	84.25	3.714915	0.0688
DeepLabv3+	79.28	83.32	5.813523	0.0381
HRNet	76.07	81.82	9.636783	0.1055
Unet	81.20	85.44	43.932931	0.0835
Proposed Model	84.28	88.59	22.077451	0.0559

**Figure 6:**
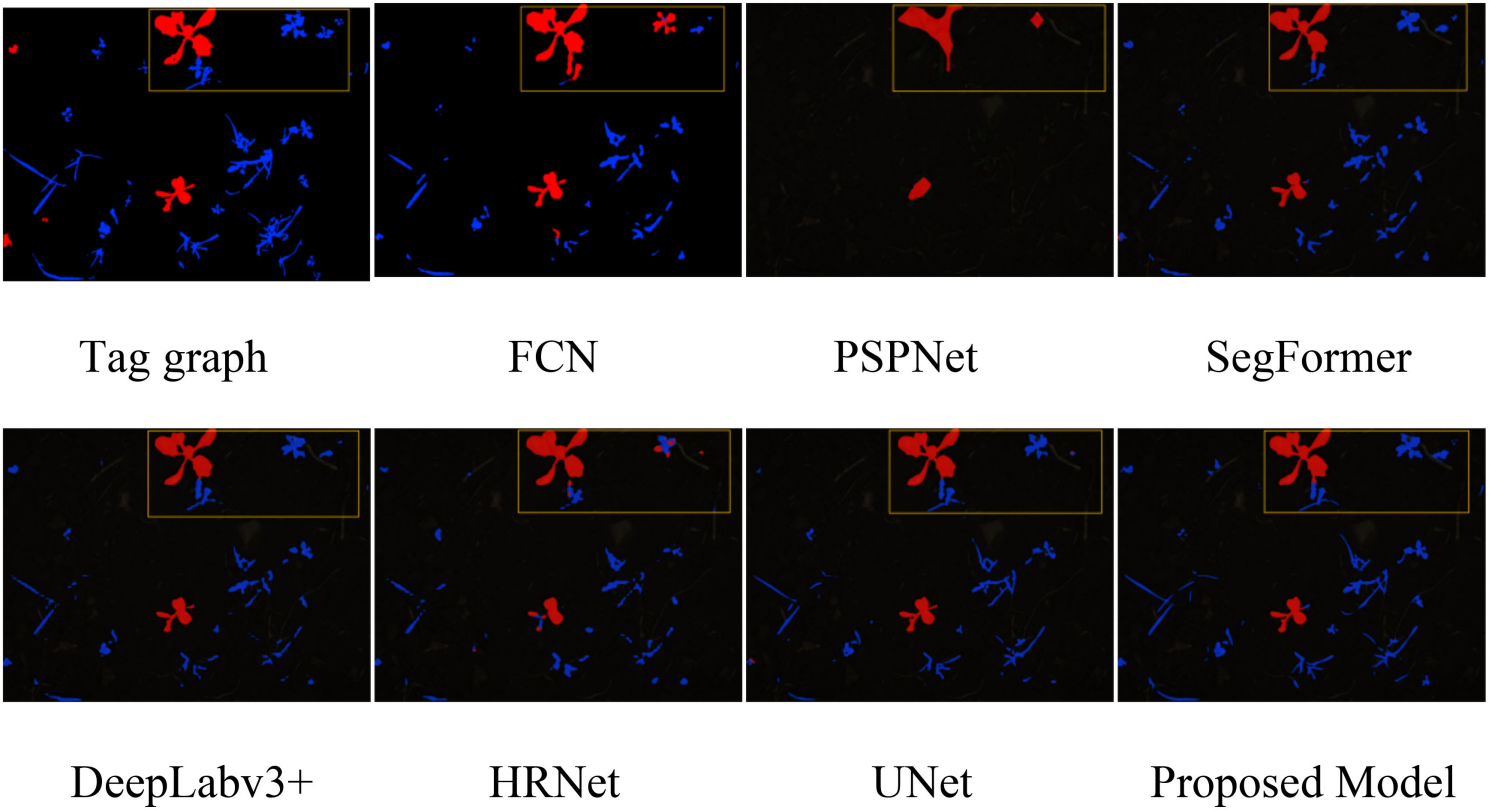
Comparison of segmentation effects of sugar beet datasets.

To visually demonstrate the performance of the proposed model, we visualized the segmentation results from the validation set on the sugar beet dataset. As shown in **Figure 6**, our model effectively optimizes edge segmentation. The red areas represent sugar beets, the blue areas represent weeds, the black areas represent soil, and the yellow boxes indicate areas where accurate segmentation was not achieved. The results show that the segmentation results of FCN, PSPNet, and HRNet are relatively coarse, especially for small targets, focusing more on crops and insufficiently on weeds, incorrectly identifying some weeds as crops. SegFormer, DeepLabv3+, and UNet show significant improvements in both primary and secondary target areas, but their edge segmentation is not accurate enough. In contrast, our model effectively addresses the aforementioned shortcomings, performing well in crop and weed segmentation, accurately capturing target details and boundaries, achieving more precise, smooth, and clear edge segmentation, and significantly improving segmentation performance in complex backgrounds with fewer errors.

### 3.3 Ablation experiments

To evaluate the impact of the MaxViT module and the CBAM attention mechanism module on the overall performance of the network, we conducted detailed ablation experiments on the sugar beet dataset. The experimental results are presented in **Table 2**. U-Net serves as the baseline model, utilizing the standard U-Net architecture. Unet_MaxViT builds upon the baseline model by replacing the U-Net backbone with the MaxViT module. Unet_CBAM extends the baseline model by incorporating the CBAM attention mechanism solely in the decoder section. The Proposed Model enhances the baseline model by substituting the backbone with MaxViT and adding the CBAM module in the decoder.

**Table 2:** The impact of different modules on the performance of the model.

Model	IoU/%			PA/%			mIoU/%	mPA/%	Params/M	Inference time/s
	soil	crop	weed	soil	crop	weed				
Unet	98.64	89.47	55.49	99.59	94.95	61.79	81.20	85.44	43.93	0.0835
Unet_MaxViT	98.89	89.45	61.58	99.62	95.14	68.80	83.31	87.85	21.82	0.0444
Unet_CBAM	98.76	89.69	60.63	99.49	95.37	70.27	83.03	88.38	44.49	0.0435
Proposed Model	98.95	90.34	63.56	99.65	95.21	70.93	84.28	88.59	22.08	0.0559

As shown in **Table 2**, the Unet model achieves IoU (Intersection over Union) values of 98.64% for soil, 89.47% for crops, and 55.49% for weeds. In terms of PA (pixel accuracy), the values for soil, crops, and weeds are 99.59%, 94.95%, and 61.79%, respectively. The mIoU and mPA are 81.20% and 85.44%, respectively, with a parameter count of 43.93M and an inference time of 0.0835s. When the backbone network is replaced with the MaxViT module, forming the Unet_MaxViT model, improvements are observed in both IoU and PA metrics. Specifically, the IoU values for soil, crops, and weeds increase to 98.89%, 89.45%, and 61.58%, respectively, while the PA values for soil, crops, and weeds rise to 99.62%, 95.14%, and 68.80%. Consequently, the mIoU and mPA improve to 83.31% and 87.85%. And the number of parameters is reduced to 21.82M, and the inference time is reduced to 0.0444s, showing higher efficiency and better performance. After incorporating the CBAM module into the U-Net model, the Unet_CBAM model demonstrated significant improvements across various metrics. Specifically, the IoU for soil, crops, and weeds increased to 98.76%, 89.69%, and 60.63%, respectively, while the PA for soil, crops, and weeds rose to 99.49%, 95.37%, and 70.27%. The mIoU and mPA improved to 83.03% and 88.38%, respectively. Although the parameter count slightly increased to 44.49M, the inference time decreased to 0.0435s, maintaining a high level of efficiency.

Finally, the Proposed Model, which incorporates the CBAM module, shows the best performance across all metrics. Specifically, the IoU values for soil, crops, and weeds are 98.95%, 90.34%, and 63.56%, respectively, while the PA values for soil, crops, and weeds are 99.65%, 95.21%, and 70.93%. The mIoU and mPA further improve to 84.28% and 88.59%. Although the parameter count slightly increases to 22.08M, the inference time remains efficient at 0.0559s. The results indicate that the introduction of the MaxViT module and the CBAM attention mechanism significantly enhances the segmentation performance and efficiency of the network, verifying the effectiveness and feasibility of the proposed method.

### 3.4 Generalization experiments

To verify the generalization of the model proposed in this paper, this paper uses two different datasets: beet dataset and sunflower dataset (Flourish Sapienza Datasets). The sunflower dataset was collected by a custom agricultural robot moving through sunflower farms in Italy. The dataset captures images taken a few days before the end of the chemical treatment period of the sunflower crop, during the crop emergence stage. It consists of 182 images annotated with three classes: crop, weed, and soil. In these images, the green areas indicate sunflowers, the red areas indicate weeds, and the black areas indicate soil. We evaluated the performance of the original UNet model and the proposed model on the sunflower dataset in terms of mIoU, mPA, parameter count, and inference time.

As shown in **Table 3**, the proposed model achieved mIoU and mPA values of 87.15% and 90.71%, respectively, representing improvements of 3.02% and 1.42% over UNet. This indicates a significant improvement in overall segmentation accuracy. The parameter count of the proposed model is 22.0777M, nearly half of UNet’s 43.9329M, demonstrating higher efficiency in computational resources and storage requirements. Although the inference time slightly increased, this trade-off is acceptable given the substantial performance improvement.

**Table 3:** The performance of the proposed model on the sunflower dataset.

Model	mIoU/%	mPA/%	Params/M	Inference time/s
Unet	84.13	89.29	43.9329	0.0125
Proposed Model	87.15	90.71	22.0777	0.0558

**Figure 7:**
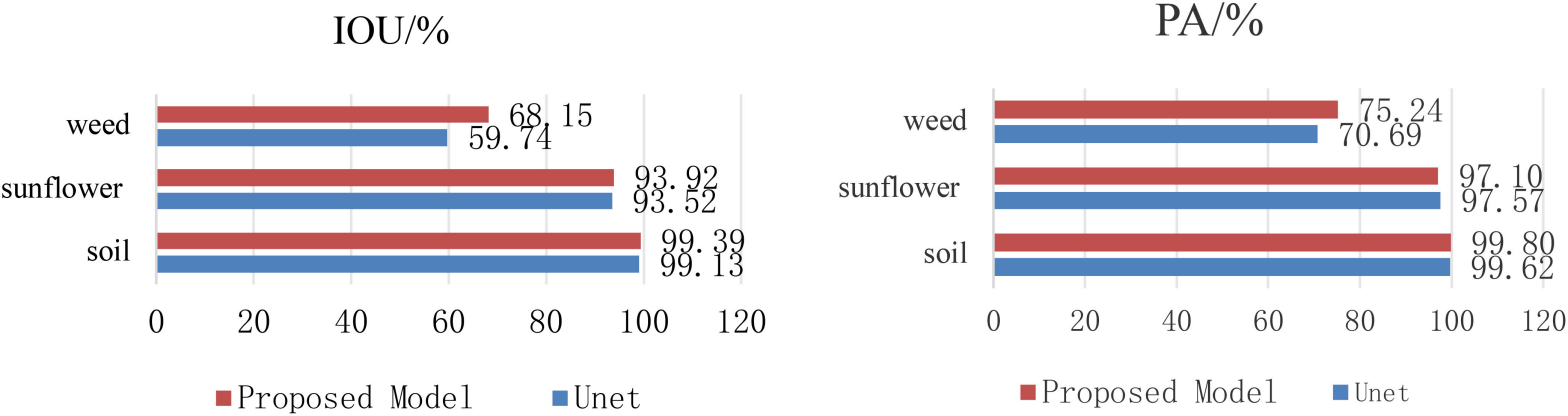
Comparison results for each category.

**Figure 7** compares the IoU and PA of the Unet model and the proposed model in each category. It can be seen from the figure that the proposed model shows higher accuracy in the segmentation of crops, weeds and soil, especially in the identification of weeds. This further proves that the proposed model has certain advantages in crop and weed segmentation.

**Figure 8:**
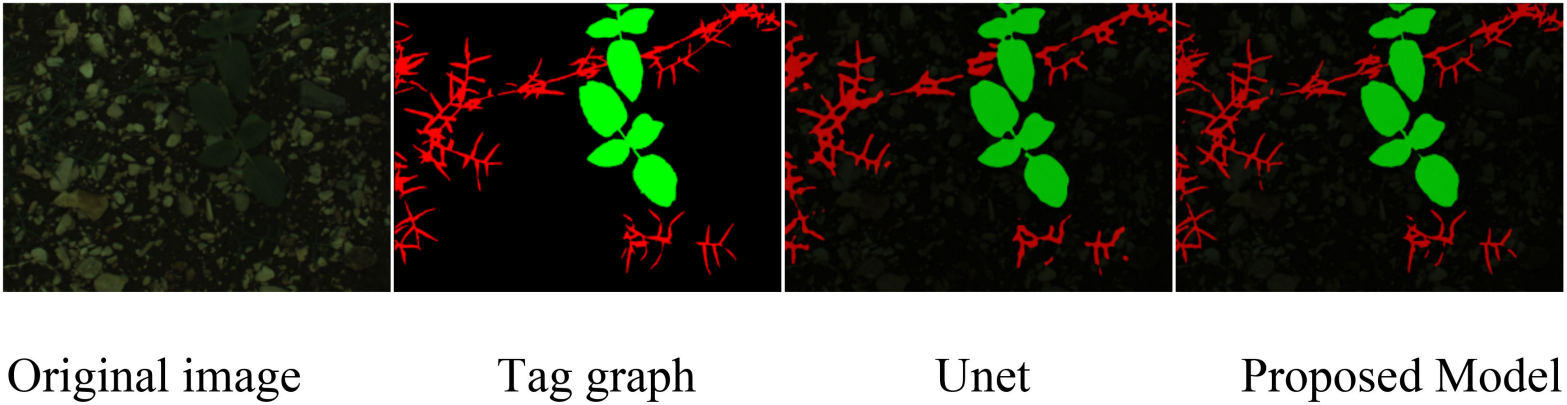
Comparison of segmentation effects of sunflower dataset.

To visually display the segmentation performance, we compared the original images, labeled images, and the segmentation results of the UNet model and the proposed model. As shown in **Figure 8**, the proposed model provides more accurate segmentation of weeds and crops, particularly in areas where weeds and soil intersect. The proposed model can better distinguish the actual boundaries of weeds, and improves the accuracy and edge clarity of weed segmentation.

Experimental results on the sunflower dataset indicate that the proposed model achieves high segmentation performance, not only excelling on the beet dataset but also performing well on the sunflower dataset. Given the distinct differences between beet and sunflower in plant size, morphology, and growth stages, the model’s incorporation of the MaxViT encoder and CBAM attention mechanism enables adaptive focus on multi-scale features, demonstrating strong adaptability. Consequently, the model achieves high segmentation accuracy across different crop types and maintains stable performance in complex field backgrounds. However, there is still room for improvement in segmentation under low-light conditions or in areas with irregular weed shapes. Overall, these results show that the proposed model exhibits strong generality and adaptability, making it suitable for image segmentation tasks across various crops and environments, and contributing to intelligent, precise agricultural production. Future research may focus on further optimizing the model structure and integrating environmental variables such as lighting and humidity to enhance the model’s adaptability and practical utility across diverse crop types and complex agricultural settings.

## 4 Conclusion

This paper proposes an efficient crop-weed segmentation model based on an improved UNet and attention mechanism. By introducing MaxViT as the encoder and combining the CBAM attention mechanism module in the decoder part, the weight of the feature map is adaptively adjusted to make the model more efficient. Focus on the edge and texture features of crops and weeds to improve segmentation accuracy. We have verified its effectiveness through a large number of experiments. The model has achieved significant performance improvements on the sugar beet data set, with mIoU reaching 84.28% and mPA reaching 88.59%, respectively improved by 3.08% compared to the traditional UNet model. and 3.15%.Additionally, compared to other mainstream semantic segmentation models (such as FCN, PSPNet, SegFormer, DeepLabv3+, and HRNet), the proposed model demonstrated clear advantages in segmentation accuracy, with an inference time of only 0.0559 seconds, showcasing its potential for real-time applications. Further analysis through ablation experiments highlighted the contribution of the MaxViT module and the CBAM attention mechanism to the overall network performance. The results showed that the inclusion of these modules significantly enhanced the model’s performance across various metrics, particularly in the segmentation of details and boundaries. Moreover, the proposed model exhibited excellent performance in generalization experiments, demonstrating good segmentation accuracy across different datasets, indicating its good robustness and versatility.

Although incorporating the MaxViT and CBAM attention mechanisms has improved segmentation accuracy, it also increases model complexity and computational demands, which may pose challenges for resource-limited devices or real-time applications. Additionally, the relatively small dataset limits the model’s generalization and robustness across diverse application scenarios. Moving forward, we aim to further optimize the model by simplifying its structure and reducing computational load while maintaining segmentation accuracy. This will make the model more lightweight and suitable for resource-constrained devices and more efficient real-time applications. Expanding the dataset’s scale and diversity is also planned, including data from various crop and weed types and testing in more complex field environments. and we plan to apply the model to real-world field platforms for testing to verify its performance in practical scenarios, taking into account challenges such as the platform’s movement speed, real-time processing, and other application-specific factors. Future research could incorporate environmental variables (e.g., climate conditions, soil moisture) and additional sensor data to enhance the model’s adaptability to diverse agricultural scenarios. By integrating more sensory data and environmental variables, we aim to further improve the model’s versatility and practical utility in intelligent agriculture.

## Data Availability

The datasets presented in this study can be found in online repositories. The names of the repository/repositories and accession number(s) can be found below: https://www.ipb.uni-bonn.de/data/sugarbeets2016/http://www.diag.uniroma1.it/~labrococo/fsd/sunflowerdatasets.html.
